# Analysis of Self-Incompatibility and Genetic Diversity in Diploid and Hexaploid Plum Genotypes

**DOI:** 10.3389/fpls.2019.00896

**Published:** 2019-07-12

**Authors:** Donia Abdallah, Ghada Baraket, Veronica Perez, Sana Ben Mustapha, Amel Salhi-Hannachi, Jose I. Hormaza

**Affiliations:** ^1^Laboratoire de Génétique Moléculaire, Immunologie et Biotechnologie, Faculté des Sciences de Tunis, Université de Tunis El Manar, Tunis, Tunisia; ^2^Unidad Técnica del IPNA-CSIC, Laboratorio de Agrobiología Juan José Bravo Rodríguez (Cabildo Insular de La Palma), Santa Cruz de La Palma, Spain; ^3^Instituto de Hortofruticultura Subtropical y Mediterránea La Mayora (IHSM La Mayora -UMA-CSIC), Algarrobo, Spain

**Keywords:** plums, polyploidy, *S*-genotyping, self-(in)compatibility, pollination, pollen tube

## Abstract

During the last decade, *S*-genotyping has been extensively investigated in fruit tree crops such as those belonging to the *Prunus* genus, including plums. In plums, *S*-allele typing has been largely studied in diploid species but works are scarcer in polyploid species due to the complexity of the polyploid genome. This study was conducted in order to analyze the *S*-genotypes of 30 diploid *P. salicina*, 17 of them reported here for the first time, and 29 hexaploid plums (24 of *P. domestica* and 5 of *P. insititia*). PCR analysis allowed identifying nine *S*-alleles in the *P. salicina* samples allocating the 30 accessions in 16 incompatibility groups, two of them identified here for the first time. In addition, pollen tube growth was studied in self-pollinated flowers of 17 Tunisian *P. salicina* under the microscope. In 16 samples, including one carrying the Se allele, which has been correlated with self-compatibility, the pollen tubes were arrested in the style. Only in one cultivar (“Bedri”), the pollen tubes reached the base of the style. Twelve *S*-alleles were identified in the 24 *P. domestica* and 5 *P. insititia* accessions, assigning accessions in 16 *S*-genotypes. *S*-genotyping results were combined with nine SSR loci to analyze genetic diversity. Results showed a close genetic relationship between *P. domestica* and *P. salicina* and between *P. domestica* and *P. insititia* corroborating that *S*-locus genotyping is suitable for molecular fingerprinting in diploid and polyploid *Prunus* species.

## Introduction

Plums, belonging to the *Prunus* genus (family Rosaceae, subfamily Prunoideae, sub-genus Prunophora), have been cultivated for at least 2000–4000 years, being among the first fruit species to attract human interest and appear to have been domesticated very early (Gautier, 1977). In fact, one of the reasons for the frequent domestication of *Prunus* species might have been the coincidence between the location of the center of diversity of *Prunus* and the first ancient high civilizations of human history (OCDE, 2002). Within Prunophora, the section Euprunus contains the Asian (such as *P. salicina* Lindl. and *P. simonii* Carr.) and European (such as *P. domestica* L., *P. cerasifera* Ehrh., *P. insititia* L. and *P. spinosa* L.) plums, whereas the section Prunocerasus contains the North American species (such as *P. americana* Marsh., *P. angustifolia* Marsh., *P. hortulana* Bailey, *P. munsoniana* Wight & Hedr., *P. maritima* Marsh. and *P. subcordata* Benth).

The basic somatic chromosome number for *Prunus* is *x* = 8. However, natural interspecific hybridization is responsible for several cases of polyploidy in this genus (Das et al., 2011). Thus, chromosome numbers of different plum species varies from diploid to hexaploid: *P. salicina, P. cerasifera, P. americana* and *P. simonii* are diploid with 2n = 16, *P. spinosa* is tetraploid with 2n = 32 whereas *P. domestica* and *P. insititia* are hexaploid with 2n = 48. Polyploidy is of widespread occurrence in plants and a major mechanism of adaptation and speciation. It is thus recognized as a major force in evolution (Van de Peer et al., 2017). Polyploidy is important, too, from a practical point of view, since polyploid plants are often more vigorous and may be more resistant to frost and the attacks of parasitic fungi than their diploid counterparts (OCDE, 2002). In contrast, the complexity of the polyploid genome makes molecular studies in these species a complex and elusive task.

Plums are mainly self-incompatible species and show, like other *Prunus* species, a Gametophytic Self-Incompatibility system (GSI). Within Rosaceae, the self-incompatibility mechanism (SI) is a widespread genetic system that promotes outcrossing by enabling hermaphrodite plants to avoid self- and cross-fertilization with close relatives. This system comprises recognition of self-related pollen, by cells of the pistil, followed by rejection of the incompatible pollen. In GSI, pollen tube growth is aborted during pollen tube growth in the style (De Nettancourt, 1977). GSI is the most common self-incompatibility system in angiosperms, and has been reported in more than 60 families of flowering plants (Kao and McCubbin, 1996). The recognition in GSI is genetically determined by a polymorphic locus (*S*), encoding at least two linked genes that determine the pistil and pollen phenotypes (Kao and Tsukamoto, 2004). In *Prunus* species, the *S*-locus is less than 70 kb (Ushijima et al., 2001) and contains two linked genes; one, *S*-RNase, is expressed in the pistil while the second, SFB (*S*-haplotype-specific F-box), is expressed in the pollen (Ushijima et al., 2001; Entani et al., 2003). Genotypes sharing the same *S*-genotypes are genetically inter-incompatible and included in the same Incompatibility Group (IG), while cultivars having different *S*-genotypes are inter-compatible and are allocated to different IG. As consequence, assignment of each cultivar to its corresponding IG is essential for orchard planning and appropriate crosses in breeding programs (Guerra and Rodrigo, 2015; Halász et al., 2017).

In the last two decades, cloning and characterization of the *S*-locus genes in species of the Rosaceae have allowed the development of fast and accurate PCR methods for *S*-allele typing based on the polymorphism of amplified fragment length. This strategy does not require controlled pollinations to evaluate the percentage of fruit set in orchard conditions or microscopic observations of pollen tube growth in flowers of controlled crosses (Guerra et al., 2009; Guerra and Rodrigo, 2015; Matsumoto and Tao, 2016). Several PCR *S*-allele typing methods (Beppu et al., 2002, 2003) were widely and successfully developed in many *Prunus* species such as Japanese plum (Yamane et al., 1999), sweet cherry (Tao et al., 1999), almond (Tamura et al., 2000), European apricot (Romero et al., 2004) and Japanese apricot (Yaegaki et al., 2001).

In Japanese plum, “Sordum” (SaSb) was the first *S*-genotyped cultivar by cloning the cDNA of the *S*-RNases in the style (Yamane et al., 1999). Since then, numerous *S*-genotyping works have been conducted using PCR and analytic and/or capillary electrophoresis to detect intron length polymorphisms of both *S*-RNase and SFB genes (Tao et al., 1999; Yamane et al., 2001; Beppu et al., 2002, 2003, 2012a; Sonneveld et al., 2003; Sapir et al., 2004, 2007; Vaughan et al., 2006; Halász et al., 2007; Zhang et al., 2007, 2008; Guerra et al., 2009, 2012; Guerra and Rodrigo, 2015). Until 2015 (Guerra and Rodrigo, 2015). A total of 42 *S*-RNase alleles and 15 SFB alleles have been identified in Japanese plum, allocating cultivars in 26 incompatibility groups (IG).

Publications regarding SI in polyploid plums are scarcer. Most of these studies were conducted to test whether polyploidy influences self-compatibility (SC). The limited works of *S*-genotyping in polyploid plums might be explained by the fact that SI occurs less frequently in polyploid than in diploid species (Halász et al., 2014). The first study regarding the molecular genetics of SI in *P. domestica* (2n = 48) was published by Sutherland et al. (2004). Later, six *S*-locus-specific markers previously used for other *Prunus* species were successfully tested on 33 domestic plum cultivars (*P. domestica*) by Kota-Dombrovska and Lācis (2013). Preliminary information regarding the *S*-genotypes of 16 *P. domestica* cultivars grown in Hungary was conducted by Halász et al. (2014). More recently, Halász et al. (2017) provided an *S*-allele profiling of 17 SI polyploid Hungarian plums (10 *P. spinosa* L., 4 *P. insititia* L. and 3 *P. spinosa* X *P. domestica*) and identified the sizes of 23 *S*-RNase alleles using PaConsII consensus primers spanning the second intron of the *S*-RNase gene.

Due to the economic importance, risk of vulnerability and the high diversity of Tunisian plum germplasm, the first objective of this study was to establish the *S*-genotypes, the incompatibility groups and the self-(in)compatibility phenotypes of 17 Tunisian *P. salicina* plums with unknown *S*-genotypes by combining PCR analysis and observation of pollen tube growth in self-pollinated flowers. The second objective was to fingerprint the *S*-alleles in hexaploid plums. Thus, 29 polyploid plums (5 *P. insititia* from Tunisia and 24 *P. domestica* accessions from Spain) with unknown *S*-genotypes were *S*-genotyped for the first time. The third objective was to elucidate the pattern of genetic diversity based on nine SSR loci. Hence, the results of *S*-locus and those of SSR markers were combined to investigate the genetic relationships between the studied *Prunus* species.

## Materials and Methods

### Plant Material

A total of 59 plum accessions were used in this study. Twenty four accessions belonged to hexaploid *P. domestica* (2n = 2x = 48), eighteen of them conserved at the CITA-Aragon germplasm collection in Zaragoza (Spain) and six collected in La Palma Island (Spain). Additionally, five accessions belonging to hexaploid *P. insititia* (2n = 2x = 48) were collected from non-cultivated populations in Tunisia. Thirty accessions belonging to diploid *P. salicina* (2n = 2x = 16) were collected from Tunisia, thirteen of them from two collections (Sodon and Sadira) and represent commercial varieties, and seventeen from growers orchards. Information related to plant material is summarized in Table 1.

**Table 1:** Names and origin of plum accessions analyzed in this work.

Species	Accession	Abbreviation	Origin	Species	Accession	Abbreviation	Origin
*P. domestica*	Alcor–1	ALC1	Germplasm collection CITA-Aragon- Zaragoza-Spain	*P. salicina*	Ain Bagra1	ANB1	Growers orchards- Tunisia
	Alcor–2	ALC2			Ain Bagra2	ANB2	
	Arenal	ARE			Ain Dhib	AND	
	Domingo	DOM			Ain Torkia	ANT	
	F–4 A–4	F4A4			AouinaHamra	ANH	
	F–9 A–10	F9A10			Aouina Hamra2	ANH2	
	Fraila	FRA			AouinaSafra	ANS	
	President	PRE			Aouina Safra2	ANS2	
	Puente Ave	PnAVE			Aouina Safra3	ANS3	
	R Claudia Conde	RCC			Bedri	BED	
	RC Aniñon	RC A			Cidre	CID	
	RC Dorada	RC D			Cidre1	CID1	
	RC Verde	RCver			Sauvage	SAU	
	Río Ribazo 1	Rio R1			Tasstour Hamra précoce	THP	
	Río Ribazo 2	Rio R2			Tasstour Hamra Tardive	THT	
	Ruth Gestteter	R Ges			Tasstour Safra	TSS	
	Tobed	TOB			Zaghwenia	ZAG	
	Verde	VER			606	606	Germplasm collections- Tunisia
	Agustina	AGU	La Palma Island-Spain		Angeleno	ANG	
	HuevoChivato	H Chi			Angeleno2	ANG2	
	Mulata	MUL			Beauty	BEA	
	Mulata1	MUL1			Black Diamant	BLD	
	Negra del paíspequeña	NPP			Black Gold	BLG	
	Negra	NEG			Black Star	BLS	
*P. insititia*	Chaaraouia	CHA	Spontaneous populations-Tunisia		Fortune	FOR	
	MeskiKbiraHamra	MKH			Marie	MAR	
	MeskiKbiraKahla	MKK			Methley	MET	
	MeskiSghiraHamra	MSH			Santa Rosa	SNR	
	Zenou	ZEN			Sungold Sungold2	SNG	
						SNG2	

### *S*-Locus Analysis

Young leaves from each of these accessions were used as the starting material to extract DNA using the cetyltrimethyl ammonium bromide (CTAB) following the protocol described by Doyle and Doyle (1987). DNA was quantified using a Nanodop 1000 and diluted to 10 ng/μL.

*S*-genotyping of plum cultivars was performed using three pairs of consensus primers. The second intron of the *S*-RNase gene was amplified using the primer pair PruC2/PCER (Tao et al., 1999; Yamane et al., 2001). The primer pairs PaCons1F/PaCons1R2 (Sonneveld et al., 2006) and Fbox5’F/Fbox–IntronR (Vaughan et al., 2006) were used to amplify the first intron of the *S*-RNase gene and the SFB gene intron, respectively.

PCRs were performed in a volume of 15 μl, with 20 mM Tris–HCl, pH 8.4, 50 mM KCl, 2 mM MgCl2, 0.1 mM of each dNTP, 0.2 μM of each primer, 40 ng of genomic DNA and 0.45 U of BioTaq^TM^ DNA polymerase (Bioline, London, United Kingdom). All PCR reactions were carried out in an I-cycler (Bio-Rad Laboratories, Hercules, CA, United States) thermocycler using the following temperature profile: an initial denaturation step at 94°C for 1 min, 35 cycles of 94°C for 3 min, 56°C for 1 min, and 72°C for 3 min. A final extension was programmed at 72°C for 7 min.

Fragments amplified with the PruC2/PCER primer combination, with sizes were greater than 500 bp, were separated using a 2% agarose gel electrophoresis, stained with SYBRGreen and visualized with UV light. Fragment size estimation was done using a size standard (1 kb DNA Ladder; Invitrogen, Carlsbad, CA, United States). Three *P. salicina* cultivars (“Fortune,” “Santa Rosa,” and “Beauty” with known *S*-genotypes (SbSc, ScSe, and SbSh, respectively) were used as references.

Fragments amplified with PaCons1F/PaCons1R2 andFbox5’F/Fbox–IntronR primers with sizes smaller than 500 bp, were analyzed by capillary electrophoresis. Forward primers were labeled with a fluorescent dye on the 5-end and PCR products were detected and sized with a Beckman Coulter Genome Lab GeXP^TM^ capillary DNA analysis system. Samples were denaturalized at 90°C for 120 s, injected at 2.0 kV for 30 s, and separated at 6.0 kV for 35 min.

### Pollination Tests

To establish self-(in)compatibility in the Tunisian diploid cultivars, 17 genotypes were self-pollinated in the laboratory and pollen tube growth was observed under the microscope.

Flowers of each genotype were collected at the balloon stage and pollen was obtained by manually removing and drying the anthers at room temperature during 24 h. The pollen was then sieved through a fine mesh and stored at −20°C until required (Guerra et al., 2009; Guerra and Rodrigo, 2015).

To obtain the pistils, flowers were collected at balloon stage 24 h before anthesis. These flowers were emasculated by removing petals, sepals and stamens with fingernails, then the pistils were maintained on wet florist foam at laboratory temperature (Rodrigo and Herrero, 1996). On the following day, a group of 25–45 flowers was hand-pollinated using a fine paintbrush. Three days later, the pollinated pistils were fixed in FAA [70% ethanol: acetic acid: formaldehyde (18 : 1 : 1, v/v/v)] (Guerra et al., 2009).

For microscope preparations, the fixed pistils were washed three times for 1 h with distilled water and left in 5% sodium sulphite at 4°C. To soften the tissues, the pistils were autoclaved at 1 kg/cm2 during 10 min in sodium sulphite (Jefferies and Belcher, 1974), and stained with 0.1% (v/v) aniline blue in 0.1 N K_3_PO_4_ to stain callose (Linskens and Esser, 1957). Pollen tube growth in the style was observed under an Olympus BH2 microscope with UV.

### SSR Analysis

The 59 plum genotypes were also analyzed using nine SSR primer pairs previously developed in *Prunus* (Supplementary Table S1). PCR reactions were carried out in a volume of 20 μl, with 20 mM Tris–HCl, pH 8.4, 50 mM KCl, 4 mM MgCl2, 0.1 mM of each dNTP, 0.2 μM of each primer, 40 ng of genomic DNA and 0.45 U of BioTaq^TM^ DNA polymerase (Bioline, London, United Kingdom). PCR reactions were run in an I-cycler (Bio-Rad Laboratories, Hercules, CA, United States) thermocycler using the following temperature cycles: an initial step of 2 min at 94°C, 35 cycles of 45 s at 94°C, 45 s at 57°C, 1 min at 72°C, and a final step of 5 min at 72°C. The obtained fragments were analyzed by capillary electrophoresis. Forward primers were labeled with a fluorescent dye on the 5-end and PCR products were detected and sized with a Beckman Coulter GenomeLabGeXP^TM^ capillary DNA analysis system. Samples were denaturalized at 90°C for 120 s, injected at 2.0 kV for 30 s, and separated at 6.0 kV for 35 min.

### Diversity Analyses

To explore the genetic diversity of the studied cultivars, different diversity parameters of the *S*-locus and SSR markers were calculated with Arlequin ver. 3.5 (Excoffier et al., 2005) for each plum species. Detected alleles for both SSR and *S*-locus markers were scored as present (1) or absent (0). Genetic distances were calculated with Maximum Composite Likelihood (Tamura et al., 2011) in Popgene ver. 1.31 software, and then imported to Mega5 to construct a UPGMA tree. Numbers on major branches represent bootstrap support from 2000 replicates. For MDS analysis, genetic distances were calculated with the Euclidean index (Taguchi and Oono, 2005) with three-dimensional coordinate system using the PAST software (Ver. 3.21) (Hammer et al., 2001).

## Results

### *S*-Locus Analysis

#### *S*-Allele Typing in Diploid *P. salicina*

**FIGURE 1:**
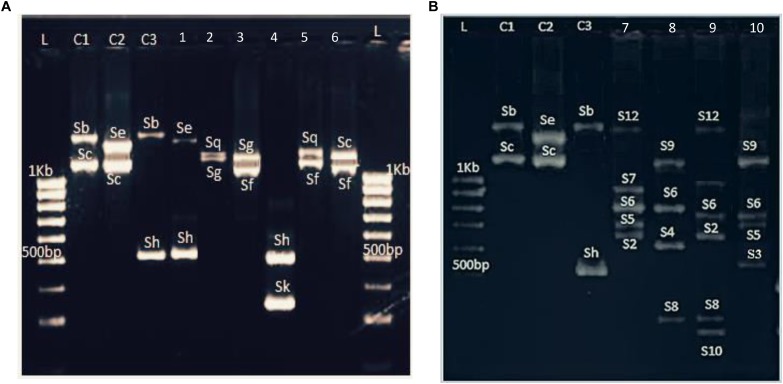
Example of *S*-allele identification in **(A)** six *P. salicina* samples and **(B)** six hexaploid plums amplified with PruC2-PCER. L: 1KB ladder. C1, C2, and C3: Varieties used as control with previously known *S*-genotypes. C1, Fortune; C2, Santa Rosa, C3, Beauty; 1, Bedri 1; 2, Aouina Hamra; 3, Tasstour Hamra précoce; 4, Sungold; 5, Ain Dhib; 6, Zaghwenia; 7, Mulata; 8, Negra; 9, Fraila; 10, Verde.

PruC2/PCER markers, spanning the second intron of the *S*-RNase gene, showed a high degree of length polymorphism. Hence, nine fragments were amplified with sizes ranging from 400 to 1600 bp (Figure 1A). The sizes of these bands were consistent with the *S*-alleles previously identified in *P. salicina*. As band sizes were larger than 500 bp, results were analyzed by agarose gel electrophoresis which make difficult to distinguish differences between some alleles with similar sizes such Sg-Sq (1250 and 1270 bp, respectively) and Sa-Sh (470 and 500 bp, respectively).

To confirm these results, a second round of amplification was conducted with the PaCons1F/PaCons1R2 primer pair, spanning the first intron of the *S*-RNase gene. Eight fragments were amplified with sizes ranged from 208 to 389 bp. Although the differentiation between Sg and Sq alleles was successful (Supplementary Table S2), it was not possible to distinguish between the Sa and Sh alleles since they showed similar sizes (388 and 389 bp).

Fbox5’F/Fbox–IntronR primer pair was additionally used to amplify the SFB gene intron. Capillary electrophoresis analysis showed nine fragments whose sizes ranged from 170 to 190 bp. Despite the reduced level of length polymorphism between bands (ranging between 1 and 2 bp), it was possible to differentiate the Sa (174 bp) and Sh (187 bp) alleles (Supplementary Table S2).

Hence, after combining the results of the three primer pairs used, nine *S*-alleles were identified in 17 Tunisian *P. salicina* genotypes of unknown *S*-allele composition (Supplementary Table S2). Additionally, the *S*-alleles of 13 *P. salicina* cultivars with previously reported *S*-genotypes were amplified in order to confirm the size of the *S*-alleles.

In all, 30 *S*-genotypes were reported, 17 of them for the first time. These cultivars have been assigned to 16 incompatibility groups (IG), three of them allocated to group 0 identified as rare genotypes and two new IG reported here for the first time (IG-F, IG-H) (Table 2).

**Table 2:** Identified *S*-genotypes and incompatibility groups for diploid *Prunus salicina*.

Variety	Detected *S*-genotype	IG	IG^∗^	References
Tasstour Safra	SfSh	A	VI	Beppu et al., 2003
Aouina Safra				
Tasstour Hamra Tardive	SfSk	B	XVI	Beppu et al., 2003
Ain Bagra1	SfSc	C	0	Beppu et al., 2003
Ain Bagra2				
Zaghwenia				
Sauvage				
Aouina Safra2				
Aouina Safra3				
Cidre	SaSe	D	0	Beppu et al., 2002
Cidre1				
Cidre2				
Tasstour Hamra précoce	SfSg	E	IX	Beppu et al., 2002
Aouina Hamra	SqSg	F	^∗^	This work
Black Diamant Black Gold Bedri1	SeSh	G	VIII	Sapir et al., 2004
Ain Dhib	SfSq	H	^∗^	This work
Angeleno	ScSh	I	VII	Sapir et al., 2004
Angeleno2				
Aouina Hamra2				
Sungold	ShSk	J	X	Halász et al., 2007
Sungold2				Zhang et al., 2007
Black Star	SeSf	K	XIII	Guerra et al., 2009
Marie				
Methley	SbSg	L	0	Beppu et al., 2003
Santa Rosa	ScSe	M	XI	Beppu et al., 2002
Beauty	SbSh	N	IV	Beppu et al., 2002
606	SaSb	O	I	Yamane et al. (1999)
Fortune	SbSc	P	II	Yamane et al. (1999)

*IG, nomenclature order used in this study for different incompatibility groups. IG^∗^nomenclature order used in previous studies. ^∗^incompatibility-groups identified only in this work.*

#### Pollination Experiments in Diploid *P. salicina*

**FIGURE 2:**
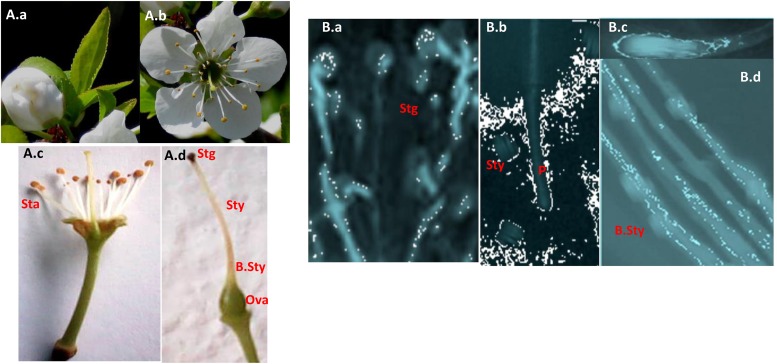
Pollen germination and pollen tube growth in flowers of Tunisian Japanese plums. **(A)** Flowers. (a) Balloon stage. (b) Full bloom. (c) Reproductive organs. (d) The pistil. **(B)** Pollen germination. (a) Germination at the stigma surface. (b) Pollen tube arrested in the style in an incompatible reaction. (c) The pollen tube tip. (d) Pollen tube growing in the base of the style in a compatible reaction. Scale bars = 50 μm. Sta, stamens; Stg, stigma; Sty, style; B.Sty, the base of the style; Ova, ovary; P, Pollen tube.

To explore self-(in)compatibility of the 17 *P. salicina* accessions analyzed, self-pollinations were carried out in the laboratory. For each cultivar, 25 to 45 flowers were collected at balloon stage (Figure 2A.a,b), emasculated (Figure 2A.c,d) and hand pollinated with the help of a paintbrush. Then, pollen tube growth in the style was observed in self-pollinated flowers under the microscope. As control, self-pollinations were tested in two cultivars: “Santa Rosa” (ScSe), known to be self-compatible, and “Fortune” (SbSc), considered as self-incompatible.

Pollen germination on the stigma was successful in the 17 cultivars tested (Figure 2B.a). In fifteen cultivars (IG-A-B-C-E-F-H-I), pollen tube growth was arrested at halfway the length of the style (Figure 2B.b,c). These self-pollination experiments confirmed the SI phenotypes of these plum accessions revealed by PCR (Table 3).

**Table 3:** Self-(in)compatibility results in 17 Tunisian plums analyzed in this work.

Cultivar	Detected *S*-genotype	Number of examined pistils	% of pistils with successful pollen germination	% of pistils with pollen tubes reaching the ovary	SI/SC
Tasstour Safra	SfSh	45	85	0	SI
Aouina Safra	SfSh	25	100	0	SI
Tasstour Hamra Tardive	SfSk	27	95	0	SI
Ain Bagra1	SfSc	27	95	0	SI
Ain Bagra2	SfSc	30	92	0	SI
Zaghwenia	SfSc	28	100	0	SI
Sauvage	SfSc	25	100	0	SI
Aouina Safra2	SfSc	25	100	0	SI
Aouina Safra3	SfSc	27	96	0	SI
Cidre	SaSe	28	100	0	SI
Cidre1	SaSe	30	96	0	SI
Cidre2	SaSe	30	99	0	SI
Tasstour Hamra précoce	SfSg	26	98	0	SI
Aouina Hamra	SqSg	26	99	0	SI
Bedri1	SeSh	45	88	75	SC
Ain Dhib	SfSq	30	95	0	SI
Aouina Hamra2	ScSh	29	90	0	SI
Fortune (C1)	SbSc	27	98	0	SI
Santa Rosa (C2)	ScSe	25	100	85	SC

As expected, in the cultivar “Bedri” (IG-G), which carries the Se allele, previously known as related to the SC phenotype (Beppu et al., 2005), pollen tubes reached the base of the style in 75% of self-pollinated flowers confirming the SC phenotype in this cultivar (Figure 2B.d). However, an unexpected result was observed in the three accessions of “Cidre” (IG-D). Pollen tube growth was arrested at halfway the length of the styles despite the identification of the Se allele by PCR, which suggested that “Cidre” is rather a self-incompatible cultivar (Table 3).

#### *S*-Genotyping in Hexaploid *P. insititia* and *P. domestica*

The three tested primer pairs exhibited successful PCR amplifications for the 29 hexaploid plum accessions (Figure 1B). Furthermore, the same fragments were amplified in both *P. insititia* and *P. domestica*.

PCR amplifications performed with PruC2/PCER showed a multiallelic profile with genotypes carrying up to six fragments (Figure 1B). The *S*-typing revealed 12 alleles with sizes ranging between 300 and 1600 bp. These alleles were named with numbers to allow distinguishing them from the alleles of diploid plums, which are named with letters (Table 4). Three alleles showed the same sizes than those reported in diploid plums: S_12_–Sb (1580 bp), S_9_–Sf (1090 bp) and S_1_–Sa (470 bp).

**Table 4:** Sizes of *S*-alleles amplified in hexaploid *P. domestica* and *P. insititia* with three primers pairs.

*S*-allele	PRUC2–PCER	PaCons1F/PaCons1R2	Fbox5’F/Fbox–IntronR
S_1_	470	374	192
S_2_	650	394	*n.a*
S_3_	580	385	189
S_4_	600	*n.a*	178
S_5_	700	377	182
S_6_	800	405	192
S_7_	900	384	177
S_8_	350	228	*n.a*
S_9_	1090	326	176
S_10_	312	350	*n.a*
S_11_	1000	413	196
S_12_	1580	368	185

*n.a, no amplification.*

To confirm these results, PCR amplifications were conducted using PaCons1F/PaCons1R2 primers. Capillary electrophoresis revealed 11 fragments with sizes ranging from 228 to 413 bp (Table 4). The use of the PaCons1F/PaCons1R2 primer pair allowed amplification of all *S*-alleles previously detected with PruC2/PCER, except the S4 allele for which no amplification with PaCons1F/PaCons1R2 was obtained. Moreover, the amplified alleles showed similar sizes between S_12_–Sb (368 bp) and S_9_–Sf (326 bp) alleles, but a difference of 15 bp was revealed between S_1_ and Sa (374 and 389 bp, respectively).

A third round of amplification was conducted using the Fbox5’F/Fbox–IntronR primer pair. Amplified bands showed small differences in length (in order of 1 or 2 bp) which required several repetitions to ensure the exact size of each band. All the *S*-alleles previously defined were detected with the Fbox5’F/Fbox–IntronR primer pair with sizes ranging between 172 and 208 bp (Table 4), except the three *S*-alleles S_2_, S_8_ and S_10_. In addition, the alleles S_12_ and S_9_ showed the same lengths than Sb (185 bp) and Sf (176 bp), respectively.

Overall, twelve *S*-alleles were detected in 29 hexaploid plums (Table 4) assigned to sixteen *S*-genotypes (Table 5).

**Table 5:** Identified *S*-genotypes and incompatibility groups for hexaploid *Prunus domestica* and *Prunus insititia*.

Variety	Detected *S*-genotype
*Prunus domestica*	
Mulata	S_2_/S_5_/S_6_/S_7_/S_12_
Mulata2	
Negra	S_4_/S_6_/S_8_/S_9_
HuevoChivato	S_2_/S_5_/S_7_/S_10_/S_8_/S_12_
Agustina	
Verde	S_3_/S_5_/S_6_/S_9_
RC Verde	
Negra del país pequeña	S_4_/S_6_/S_11_/S_9_
President	S_1/_S_5_/S_7_/S_9_
Ruth Gestteter	S_1_/S_3_/S_5_/S_6_/S_12_
RC Dorada	S_1_/S_3_/S_2_/S_11_/S_9_
Fraila	S_2_/S_8_/S_10_/S_6_/S_12_
Alcor–1	S_1_/S_3_/S_6_/S_9_
Alcor–2	
Arenal	
Domingo	
F–9 A–10	
Puente Ave	
R Claudia Conde	
RC Aniñon	
Río Ribazo 1	
Río Ribazo 2	
Tobed	
F–4 A–4	S_1/_S_3_/S_5_/S_11_/S_9_
*Prunus insititia*	
Meski Kbira Kahla	S_6_/S_7_/S_8_/S_10_
Meski Sghira Hamra	S_6_/S_7_/S_10_/S_11_
Chaaraouia	S_7_/S_8_/S_10_/S_11_
Meski Kbira Hamra	S_6_/S_5_/S_8_/S_10_
Zenou	S_1_/S_2_/S_10_/S_11_

#### Genetic Diversity at the *S* Locus

Using the three *S*-markers, nine *S*-alleles have been identified in 30 diploid *P. salicina* cultivars; among them, the Sf allele was the most frequent (0.22). In hexaploid plums, among 12 *S*-alleles, S_1_ (0.19) and S_10_ (0.21) were the most frequent in *P. domestica* and *P. insititia*, respectively (Table 5). In *P. salicina*, allelic richness (*A*r) ranged between 0.11 and 0.48 with an average of 0.3. In *P. domestica*, *A*r ranged between 0.35 and 0.49 with an average of 0.42, while in *P. insititia* it ranged between 0.37 and 0.51 with an average of 0.44 (Table 6).

**Table 6:** Diversity parameters of tested *Prunus* species based on *S*-locus markers.

Group	n	Genetic diversity					Heterozygosity			F_ST_		
		A	MAF	*A*r	Gn	Gd	*H*o	*H*e	*f*	*P. salicina*	*P. domestica*	*P. insititia*
*P. salicina*	30	9	0.22	0.3	16	0.77	0.87	0.74	−0.18	–	0.18^∗^	0.23
*P. domestica*	24	12	0.19	0.42	11	0.86	0.86	0.75	−0.15	0.18^∗^	–	0.16^∗^
*P. insititia*	5	8	0.21	0.44	5	1	0.86	0.75	−0.15	0.23	0.16^∗^	–
Average	59	9.66	0.2	0.39	10.6	0.88	0.86	0.75	−0.15		0.19	

*n, number of accessions per group; A, total number of S-alleles; MAF, major allele frequency; Ar, Allelic richness; Gn, total number of S-genotypes; Gd, genotypic diversity; Ho, observed heterozygosity; He, expected heterozygosity; f, Wright’s fixation index; F_ST_, The differentiation index.*

A total of 32 *S*-genotypes were identified, 16 of them belonging to *P. salicina*, 11 to *P. domestica* and 5 to *P. insititia*. Genotypic diversity (Gd) showed high values and ranged between 0.54 and 1 in *P. salicina* (an average of 0.77), 0.72 and 1 in *P. domestica* (an average of 0.86) and 1in *P. insititia* (Table 6). This Gd depends on allele frequency in each genotype (whether the allele is frequent or rare) and on the number of accessions sharing the same genotype.

These high GD reflected an important frequency of the heterozygous fraction. The observed heterozygosity (*H*o) ranged from 0.8 to 0.94 in diploid plums with an average of 0.87. In hexaploid plums, *H*o ranged between 0.83 and 0.91 with an average of 0.86 for both *P. domestica* and *P. insititia*. These values were higher than the expected heterozygosities (*H*e), which ranged between 0.71 and 0.78 in diploid plums with an average of 0.74 and between 0.74 and 0.77 in hexaploid plums with an average of 0.75. Consequently, negative values of Wright’s fixation index were observed in *P. salicina* (*f* = −0.18) as well as in hexaploid plums (*f* = −0.15) (Table 6).

The differentiation index (*F*_ST_) between the three groups was 0.19. *F*_ST_ values were statistically significant between *P. domestica* and *P. salicina a*nd between *P. insititia* and *P. domestica* (0.18, 0.16, respectively with *P* < 0.05); however, F_ST_ values were not significant between *P. salicina* and *P. insititia* (0.23, 0.05 < *P* < 0.1) (Table 6). In fact, a value of F_ST_ ranging between 0.15 and 0.25 indicated that the analyzed populations are genetically different, independently from their geographic origins (Weir and Cockerham, 1984).

### SSR Analysis

The nine SSR markers proved to be highly polymorphic in the three species tested. The BPPCT012 primer pairs amplified the highest number of alleles while the UDP96-008 amplified the lowest number of alleles in the different species.

In diploid *P. salicina*, 94 alleles (*A*o) were amplified in the 30 accessions resulting in a value of 10.5 alleles per locus (*A*l) (Table 7). A total of 33.1 of them represent effective alleles (*A*e) indicating that an important number of alleles appeared at low frequency (less than 5%) which explains the high allelic richness observed (*A*r = 0.78). The loci analyzed allowed identifying 24 genotypes (Table 7).

**Table 7:** Diversity parameters of tested *Prunus* species based on SSR loci.

Species	n	Genetic diversity					Heterozygosity		
		*A*o	*A*l	*A*e	*A*r	Gn	*H*o	*H*e	*f*
*P. salicina*	30	94	10.5	33.1	0.78	24	0.71	0.67	−0.06
*P. domestica*	24	195	21.6	50.4	0.82	20	0.73	0.69	−0.06
*P. insititia*	5	73	8.1	29.3	0.74	5	0.74	0.7	−0.06

*n, number of accessions per group; Ao, number of effective alleles; Al, number of alleles per SSR locus; Ae, number of effective alleles; Ar, Allelic richness; Gn, total number of S-genotypes; Ho, observed heterozygosity; He, expected heterozygosity; f, Wright’s fixation index.*

In hexaploid *P. domestica*, the number of amplified alleles per genotype ranged from 1 to 6. The used SSR markers amplified 195 alleles (*A*o) giving a value of 21.6 alleles per locus (*A*l) with 50.4 effective alleles (*A*e) leading to an important allelic richness (*A*r = 0.82). With the whole set of nine SSRs used it was possible to identify 20 different genotypes among the 24 cultivars studied (Table 7).

In hexaploid *P. insititia*, 73 alleles were amplified (*A*o) in the 5 tested cultivars with an average of 8.1 alleles per locus (*A*l). A total of 29.3 were defined as effective alleles (*A*e) reflecting the high allelic richness (*A*r = 0.73). The five accessions had different genotypes using the whole set of SSR markers (Gn = 5) (Table 7).

In the three tested species, observed heterozygosity values were higher than those of expected heterozygosity leading to negative values of *f* (−0.06). In *P. salicina*, the observed heterozygosity ranged between 0.65 and 0.78 with an average of 0.71, whilst the expected heterozygosity ranged between 0.61 and 0.73 with an average of 0.67. In *P. domestica*, the observed heterozygosity varied from 0.71 to 0.76 with an average of 0.73, while the frequency of expected heterozygosity ranged between 0.66 and 0.73 with an average of 0.69. In *P. insititia*, the average of observed heterozygosity was 0.74 and the expected heterozygosity was 0.7 (Table 7).

#### Genetic Relationships Among the Accessions

**FIGURE 3:**
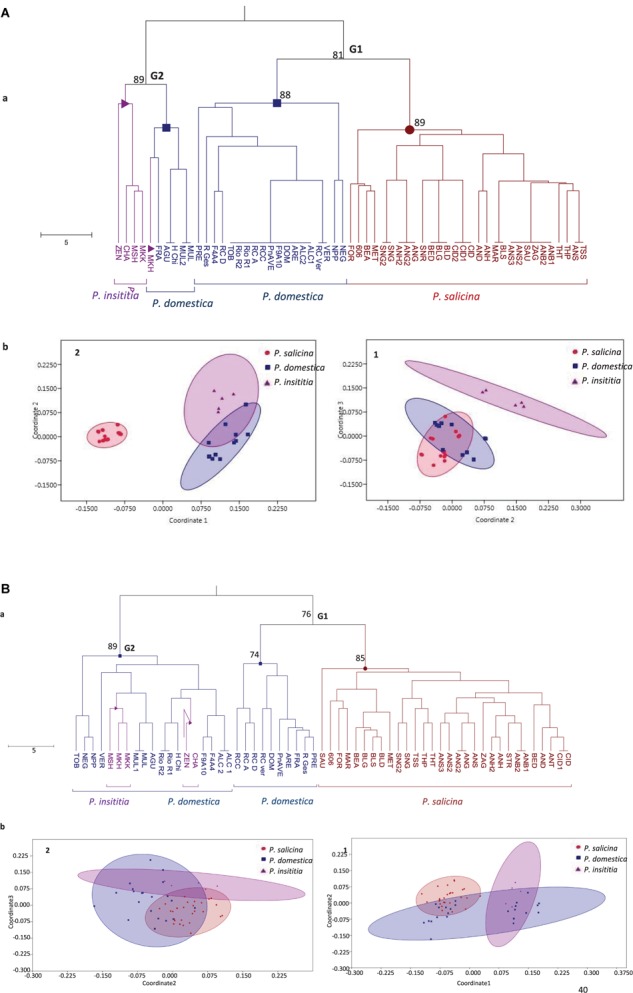
Genetic relationships between the 59 tested plums based on **(A)** *S*-locus data and **(B)** SSR data. (a) UPGMA tree. Numbers on major branches represent bootstrap support from 2000 replicates. (b) Non-metric multidimensional scaling MDS plots: (1) using dimensions 2–3. (2) using dimensions 1–2.

Based on the defined *S*-alleles, the constructed UPGMA tree divided the cultivars into two heterogeneous groups (Figure 3A.a). The first group (G1) clustered together Japanese plums (*P. salicina*) with some European plums (*P. domestica*). This result suggests a close genetic relationship between *P. salicina* and *P. domestica*. The second group (G2) grouped the remaining *P. domestica* accessions together with *P. insititia* plums.

Multidimensional scatter plots (MDS) yielded similar results than the UPGMA tree (Figure 3A.b). Using dimensions 2 and 3, the first MDS plot (1) clearly separated *P. insititia* accessions from *P. domestica* and *P. salicina*, and confirmed a close genetic relationship between *P. domestica* and *P. salicina*. In contrast, using dimensions 1 and 2, the second MDS plot (2) showed a close relationship between European plums (*P. domestica* and *P. insititia*), while Japanese plums (*P. salicina*) were relatively separated.

Based on SSR loci, the UPGMA dendrogram (Figure 3B.a) showed the same topology than that constructed with *S*-markers and clearly divided the tested accessions into two main groups: G1 and G2. The first group (G1) gathered *P. salicina* and *P. domestica* accessions. The first sub-group in that main group clustered the diploid *P. salicina* accessions and the second sub-group gathered 10 *P. domestica* accessions. The remaining *P. domestica* accessions and the five *P. insititia* cultivars fell into the second group (G2).

In addition, MDS plots were drawn based on these SSR markers. Using dimensions 1 and 2, the first MDS plot (1) confirmed the close genetic relationship between *P. salicina* and *P. domestica* and between *P. domestica* and *P. insititia.* Using dimensions 2 and 3, the MDS plot (2) showed an overlap between the three species (Figure 3B.b).

## Discussion

This study was conducted in order to report the *S*-genotypes of 59 plum accessions covering two levels of ploidy: diploid *P. salicina* (2n = 16) and hexaploid *P. domestica* and *P. insititia* (2n = 46). Among them, thirteen *P. salicina* accessions were previously *S*-genotyped and 46 accessions were analyzed for the first time. In addition, *S*-locus and SSR data were combined to investigate the genetic relationships between the species studied.

### *S*-Locus Analysis

#### Applicability of Intron Length Polymorphism Technique (ILP) in Diploid and Hexaploid Plums

The ILP technique, the most common method for *S*-allele profiling in *Prunus* species (Sonneveld et al., 2003), was used with three *S*-locus specific consensus primer pairs. Two primer pairs amplified the first and second introns of the *S*-RNase coding region and one primer pair amplified the SFB intron. These markers were developed and used earlier to study SI in several *Prunus* species (Ushijima et al., 1998; Tao et al., 1999; Sutherland et al., 2004; Sonneveld et al., 2006; Vaughan et al., 2006; Guerra et al., 2009; Herrera et al., 2018) and showed good applicability for plum germplasm characterization and cultivar discrimination which confirmed the high conservation of *S*-genes among *Prunus* species and primer pair transferability among species (Kota-Dombrovska and Lācis, 2013).

PruC2/PCE-R, spanning the second intron of the *S*-RNase gene, amplified up to six fragments in *P. salicina*, *P. insititia* and *P. domestica*. Although it has been suggested that primers specific to the second intron of *S*-RNase gene were not suitable for *S*-genotyping (Sutherland et al., 2007), this primer pair was sufficient for successful genotyping in the species tested.

Primer pairs PaCons1F/PaCons1R2, specific to the first intron of the *S*-RNase gene, and Fbox5’F/Fbox–IntronR, spanning the SFB gene intron, showed limited polymorphism and amplified alleles with similar sizes, which make the amplified fragments hardly distinguishable on agarose gel electrophoresis. However, these markers should be employed to ensure a more accurate characterization. When amplification results are analyzed by capillary electrophoresis, these primers can be used as supplementary markers in the characterization of plum genetic resources.

#### Self-Compatibility in Japanese Plums

Pollination experiments provided complementary information to the PCR amplification in order to establish the self-(in)compatibility phenotypes of Tunisian diploid plums. Although Japanese plum is known to be SI, the SC reversion is not excluded in this species as in other *Prunus* species. The SC in species of this genus has been associated to particular *S*-alleles; examples include apricot (Vilanova et al., 2006; Halász et al., 2014; Herrera et al., 2018), almond (Fernandez i Marti et al., 2014; Kodad et al., 2015), peach (Tao et al., 2007; Hanada et al., 2014), sour cherry (Yamane et al., 2003) and sweet cherry (Wünsch and Hormaza, 2004; Cachi and Wünsch, 2014). In *P. salicina*, the SC was firstly related to the Se haplotype expression (Beppu et al., 2005, 2010; Guerra et al., 2009; Guerra and Rodrigo, 2015), for which an accumulation of insufficient levels of Se-RNase due to very low transcription levels in the pistil has been described (Watari et al., 2007; Guerra et al., 2009). After that, other *S*-haplotypes have been identified as SC related, such as Sb (Guerra et al., 2009; Beppu et al., 2010), St (Beppu et al., 2012a) and Sg (Beppu et al., 2012b).

Pollen tube growth and PCR analysis showed that “Bedri,” carrying the Se allele, is a SC cultivar, which may confirm the implication of this allele in SC. In contrast, three accessions of “Cidre,” also carrying the Se allele, were revealed as SI. Similar results were observed in previous studies where some Japanese plum cultivars, described as SI, which carried one of the alleles related to the SC in their *S*-genotypes (Beppu et al., 2002, 2003; Guerra et al., 2009; Guerra and Rodrigo, 2015). Thus, SC in Japanese plum could be also related to a double phenotypic expression of the same allele, as it has been reported in almond (Fernandez i Marti et al., 2010, 2014), than associated uniquely with specific *S*-alleles (Guerra and Rodrigo, 2015).

#### *S*-Genotyping in Hexaploid Plums

Sixteen *S*-genotypes were assigned for polyploid individuals. Only one genotype carrying six alleles (IG-3) was defined, while the remaining *S*-genotypes carried four or five alleles. Similar results were observed using different molecular approaches (*S*-genotyping and SSR markers) in polyploid *Prunus* species such *P. domestica*, *P. spinosa*, and *P. insititia* (Kota-Dombrovska and Lācis, 2013; Halász et al., 2014, 2017; Urrestarazu et al., 2018; Baraket et al., 2019). These incomplete *S*-genotypes in some accessions can be the consequence of the multiple appearance of a given allele (Walsh et al., 1992; Kota-Dombrovska and Lācis, 2013). This homozygosity leads to the question of whether polyploidy can cause SC in these species. Hence, direct association between polyploidy and SC has not been observed in some polyploid *Prunus* species. In the allotetraploid *P. cerasus*, the heteroallelic pollen retained its SI phenotype and SC was related to the co-expression of two non-functional *S*-haplotypes in the same genotype (Hauck et al., 2006). Additionally, polyploidy seemed not to cause SC in *P. spinosa* and *P. insititia* (Nunes et al., 2006). In contrast, polyploidy influences SC in Solanaceae and Scrophulariaceae (Mable, 2004; Nunes et al., 2006; Vieira et al., 2008).

In this work, an average of 0.41 alleles per polyploid accession was reported using three *S*-markers. This number is relatively low for species whose allopolyploid genome structure tends to increase the number of alleles and promote the emergence of new alleles (Ainouche and Wendel, 2014; Halász et al., 2017). Additionally, 23 *S*-alleles were assigned in 17 polyploid plums in Halász et al. (2017). This result may be explained by the low heterogeneity of our tested samples. Halász et al. (2017) used the PaConsII primer pair, spanning the second intron of the *S*-RNase gene. Compared to the result of PruC2/PCE-R specific to the same region, four *S*-alleles (S_2_, S_4_, S_6_, and S_11_) showed sizes similar with S_R_, S_Z_, S_S_, and S_J_, respectively (Halász et al., 2017). In addition, four alleles (S_1_, S_3_, S_5_, and S_9_) showed differences less than 20 bp with S_O_, S_B_, S_D_, and S_M_, respectively, and two alleles (S_7_ and S_12_) showed differences less than 80 bp with S_I_ and S_V_, respectively (Halász et al., 2017).

### SSR Polymorphism

The level of SSR polymorphism observed in 30 *P. salicina* (10.5) is comparable to that revealed in 29 *P. salicina* accessions using eight SSR loci (12) by Carrasco et al. (2012). This variability is higher than that reported for *P. salicina* in other works, i.e., Mnejja et al. (2004) and Klabunde et al. (2014). It is also higher than that observed in other *Prunus* species such as almond (Fathi et al., 2008), cherry (Wünsch and Hormaza, 2002; Baris et al., 2017) and apricot (Gürcan et al., 2015).

Due to their polyploid genomes, few studies have focused on evaluating the genetic diversity of hexaploid plum species (Horvath et al., 2011; Sehic et al., 2015; Halász et al., 2017; Urrestarazu et al., 2018). A high degree of polymorphism has been revealed in hexaploid plums in previous works (Urrestarazu et al., 2018).

In general, among the *Prunus* genus, plums are considered as one of the most polymorphic species (with almond) (Salazar et al., 2014) due to their SI system. In SI populations, the fact that self-fertilization is avoided decreases the probability of obtaining homozygote offspring (Balloux, 2004; Stoeckel et al., 2006) and tends to increase the genetic diversity as well as heterozygosity levels elsewhere in the genome (Hartl and Clark, 1997; Stoeckel et al., 2006).

### Genetic Richness and Allelic Singularity of Tunisian Plums

The characterization of 17 Tunisian *P. salicina* plums has allowed assigning them into nine IG (IG-A→IG-I). Three *S*-genotypes, previously defined as rare genotypes and included under IG-0 (Beppu et al., 2003; Guerra et al., 2009; Guerra and Rodrigo, 2015) were amplified in ten accessions and assigned to IG C, D, and L. Additionally, two new genotypes (IG F and H) were reported here for the first time. SSR results showed a significant genetic diversity in the *P. salicina* tested genotypes. These results highlight the allelic singularity and the rich variability characterizing the Tunisian plum germplasm as an unexplored source of genetic variation. In fact, several Tunisian *Prunus* varieties have disappeared during the last years due to the lack of programs addressed to preserve these local germplasm (Nabli, 2011; Abdallah et al., 2016; Baraket et al., 2019), which require the establishment of appropriate germplasm collections. The results obtained in this study could complement previous work of Baraket et al. (2019) in which 23 Tunisian plums accessions were phenotypically and genetically characterized.

### Genetic Relationships in *Prunus* Species

Phylogenetic tree and Multidimensional scatter plots (MDS) for both *S*-locus and SSR loci underlined genetic relationships between the three plum species studied, especially between *P. domestica* and *P. salicina* and between *P. domestica* and *P. insititia*. Similar results were reported in previous works such in Hegedus and Halasz (2006) where the same alleles were reported in *P. domestica*, *P. domestica* × *P. cerasifera* hybrids, *P. spinosa* and *P. salicina*.

The close genetic relationship found between *P. domestica* and *P. insititia* is expected. In fact, *P. insititia* has been reported in wild form in three continents (southern Europe, Western Asia and northern Africa) (Malcolm, 2006). Although the ancestral form of this species remains still unknown, *P. insititia* has often been proposed to be a subspecies of *P. domestica* and chloroplast DNA analysis seems to corroborate this hypothesis (Reales et al., 2010).

The origin of *P. domestica* has also been debated for over a century (Crane and Lawrence, 1929; Bajashvili, 1991; Eryomine, 1991; Zohary, 1992; Hegedus and Halasz, 2006; Liu et al., 2007; Zhebentyayeva et al., 2019). The most accepted hypothesis is that of Crane and Lawrence (1929) in which *P. domestica* would be an allopolyploid hybrid species between tetraploid *P. spinosa* (2n = 2x = 32) and diploid *P. cerasifera* (2n = 2x = 16). Thus, *P. domestica* is believed to have arisen in the Caucasian region where these species are originated (Crane and Lawrence, 1929) via either chromosome doubling of the hybrid triploid or as a product of unreduced gametes from both parents (Liu et al., 2007). Bajashvili (1991) suggested that European plum could have also been originated directly from hexaploid *P. divaricata* (=*P. cerasifera*) or from *P. spinosa* by alloploidization. Moreover, Zohary (1992) reported that comparative morphology and available cytogenetic evidence do not support the assumption that *P. spinosa* contributed its two genomes (or even a single genome) to hexaploid *P. domestica* and suggested that *P. domestica* would be an autopolyploid *P. cerasifera* (Zohary, 1992). Eryomine (1991) proposed that *P. domestica* originated by artificial selection by early Eurasian societies and that several species participate in the origin of *P. domestica* probably explaining why *P. domestica* has never been found in the wild. He suggested that hybrids between (*P. cerasifera* × *P. salicina*) × *P. spinosa* and (*P. cerasifera subsp. macrocarpa*) presented the highest morphological similarities with *P. domestica*. In addition, *P. cerasifera* (CC) × *P. salicina* (SaSa) hybrids developed unreduced gametes (Eryomine, 1991). Hence, the genotypic formula suggested by Eryomine (1991) for *P. domestica* is SSCCCSa, where part S represents the genome of *P. spinosa*, part C represents the genome of diploid *P. cerasifera* and part Sa represents the genome of diploid *P. salicina* inherited into domestic plum. Recent results seem to support the hypothesis that *P. domestica* could be a hybrid of interspecific hybrids including *P. cerasifera*, *P. spinosa*, and other Eurasian plum species (Zhebentyayeva et al., 2019). Additionally, the close genetic relationship between *P. domestica* and *P. salicina* obtained by *S*-locus and SSR markers may represent an argument in favor of Eryomine proposal and may confirm that diploid *P. salicina* contributed to *P. domestica* formation.

## Conclusion

The main objective of this study was to analyze the *S*-genotypes of 30 diploid *P. salicina* and 29 hexaploid plums (24 of *P. domestica* and 5 of *P. insititia*). The results obtained are relevant at three levels: (i) The identification of new *S*-alleles and new *S*-genotypes in the *Prunus* species analyzed should facilitate future work focused on exploring and understanding the SI system in diploid and, especially, in polyploid *Prunus* species. (ii) The identification of *S*-genotypes and the genetic characterization of Tunisian local plums showed that the Tunisian germoplasm could represent a new source of variability useful in future breeding programs. In addition, such results could be used for defining a Tunisian core collection aiming to preserve the extant diversity in local Tunisian plums. (iii) Combined with SSR markers, the information obtained from the *S*-locus may supply additional information regarding the genetic relationships among *Prunus* species.

## Author Contributions

DA conducted the experiments and statistical analyses, developed the genetic analyses, and wrote the manuscript. GB provided some plant material, and discussed and corrected the content. VP provided some plant material and discussed the results. SBM conducted the pollination experiments and the microscope observations. AS-H and JH provided experimental instructions, supervised the work, and assisted in writing the manuscript.

## Conflict of Interest Statement

The authors declare that the research was conducted in the absence of any commercial or financial relationships that could be construed as a potential conflict of interest.
