# Supplementary material for: Analysis of Self-Incompatibility and Genetic Diversity in Diploid and Hexaploid Plum Genotypes
Source: Front Plant Sci. 2019 Jul 12;10:896. doi: 10.3389/fpls.2019.00896 (PMC6640205; doi:10.3389/fpls.2019.00896)
Supplement: Supplementary file 1 [file Table_1.docx]

Supplementary Table 1. Characteristics of the nine SSR primers used.

| **SSR loci** | **Linkage Group** | **Genetic Position (cM)*** | **Repeat motif** | **Sequences of SSR primers** | **Size range (bp)** | **Reference** |
| --- | --- | --- | --- | --- | --- | --- |
| BPPCT028 | LG1 | 77.4 | (TC)_15_ | F: TCAAGTTAGCTGAGGATCGC  R: GAGCTTGCCTATGAGAAGACC | 148-183 | Dirlewanger et al., 2002 |
| UDP96-005 | LG1 | 29.2 | (AC)_16_TG(CT)_2_CA(CT)_11_ | F: GTAACGCTCGCTACCACAAA  R: CACCCAGCTCATACACCTCA | 110-170 | Testolin et al., 2000 |
| BPPCT002 | LG2 | 20.9 | (AG)_25_ | F: TCGACAGCTTGATCTTGACC  R: CAATGCCTACGGAGATAAAAGAC | 196-235 | Dirlewanger et al., 2002 |
| BPPCT007 | LG3 | 11.2 | (AG)_22_(CG)_2_(AG)_4_ | F: TCATTGCTCGTCATCAGC  R: CAGATTTCTGAAGTTAGCGGTA | 118-154 | Dirlewanger et al., 2002 |
| UDP96-008 | LG3 | 36.4 | (CA)_23_ | F: TTGTACACACCCTCAGCCTG  R: TGCTGAGGTTCAGGTGAGTG | 142-161 | Cipriani et al., 1999 |
| BPPCT010 | LG4 | 2.1 | (AG)_4_GG(AG)_10_ | F: AAAGCACAGCCCATAATGC  R: GTACTGTTACTGCTGGGAATGC | 103-143 | Dirlewanger et al., 2002 |
| PMS3 | LG4 | 10.7 | CT, CA and GA | F: TGGACTTCACTCATTTCAGAGA  R: ACTGCAGAGAATTTCACAACCA | 129-165 | Struss et al., 2003 |
| BPPCT012 | LG8 | 24.1 | (CT)_13_CC(CT)_7_ | F: ACTTCCATTGTCAGGCATCA  R: GGAGCAACGATGGAGTGC | 127-193 | Dirlewanger et al., 2002 |
| UDP98-409 | LG8 | 42 | CT and GT | F: GCTGATGGGTTTTATGGTTTTC  R: CGGACTCTTATCCTCTATCAACA | 119-153 | Cipriani et al., 1999 |

*****Genetic Position of the SSR loci in centimorgan (cM) in the *Prunus*-TE-F2 linkage map (<http://www.rosaceae.org/peach/genome>).
