# Supplementary material for: Analysis of Self-Incompatibility and Genetic Diversity in Diploid and Hexaploid Plum Genotypes
Source: Front Plant Sci. 2019 Jul 12;10:896. doi: 10.3389/fpls.2019.00896 (PMC6640205; doi:10.3389/fpls.2019.00896)
Supplement: Supplementary file 2 [file Table_2.docx]

Supplementary Table 2. Sizes of *S-*alleles amplified in diploid *P. salicina* with three primers pairs.

| ***S-*allele** | **PRUC2–PCER** | **PaCons1F/PaCons1R2** | **Fbox5’F/Fbox–IntronR** | **Reference** |
| --- | --- | --- | --- | --- |
| Sa | 460 | 389 | 174 | Beppu et al., 2002 ; 2003 |
| Sb | 1580 | 368 | 185 | Beppu et al., 2002 ; 2003 |
| Sc | 1160 | 343 | 188 | Beppu et al., 2002 ; 2003 |
| Se | 1440 | 373 | 186 | Beppu et al., 2002 ; 2003 |
| Sf | 1090 | 326 | 176 | Beppu et al., 2002 ; 2003 |
| Sg | 1250 | *n.a* | 190 | Beppu et al., 2002 ; 2003 |
| Sh | 500 | 388 | 187 | Beppu et al., 2002 ; 2003 |
| Sk | 390 | 384 | 184 | Beppu et al., 2002 ; 2003 |
| Sq | 1270 | 208 | 191 | Guerra et al., 2009 |

**Note**: *n.a*. no amplification
